# Secretome Profiling by Proteogenomic Analysis Shows Species-Specific, Temperature-Dependent, and Putative Virulence Proteins of *Pythium insidiosum*

**DOI:** 10.3390/jof8050527

**Published:** 2022-05-20

**Authors:** Theerapong Krajaejun, Thidarat Rujirawat, Tassanee Lohnoo, Wanta Yingyong, Pattarana Sae-Chew, Onrapak Reamtong, Weerayuth Kittichotirat, Preecha Patumcharoenpol

**Affiliations:** 1 Department of Pathology, Faculty of Medicine, Ramathibodi Hospital, Mahidol University, Bangkok 10400, Thailand; 2 Research Center, Faculty of Medicine, Ramathibodi Hospital, Mahidol University, Bangkok 10400, Thailand; thidarat.ruj@mahidol.ac.th (T.R.); tassanee.loh@mahidol.ac.th (T.L.); wanta.yin@mahidol.ac.th (W.Y.); pattarana.sae@mahidol.ac.th (P.S.-C.); 3 Department of Molecular Tropical Medicine and Genetics, Faculty of Tropical Medicine, Mahidol University, Bangkok 10400, Thailand; onrapak.rea@mahidol.edu; 4 Systems Biology and Bioinformatics Research Group, Pilot Plant Development and Training Institute, King Mongkut’s University of Technology Thonburi, Bangkhuntien, Bangkok 10150, Thailand; weerayuth@gmail.com; 5 Interdisciplinary Graduate Program in Bioscience, Faculty of Science, Kasetsart University, Bangkok 10900, Thailand; yumyai@gmail.com

**Keywords:** *Pythium insidiosum*, pythiosis, proteome, genome, secretome, pathogenicity

## Abstract

In contrast to most pathogenic oomycetes, which infect plants, *Pythium insidiosum* infects both humans and animals, causing a difficult-to-treat condition called pythiosis. Most patients undergo surgical removal of an affected organ, and advanced cases could be fetal. As a successful human/animal pathogen, *P. insidiosum* must tolerate body temperature and develop some strategies to survive and cause pathology within hosts. One of the general pathogen strategies is virulence factor secretion. Here, we used proteogenomic analysis to profile and validate the secretome of *P. insidiosum*, in which its genome contains 14,962 predicted proteins. Shotgun LC–MS/MS analysis of *P. insidiosum* proteins prepared from liquid cultures incubated at 25 and 37 °C mapped 2980 genome-predicted proteins, 9.4% of which had a predicted signal peptide. *P. insidiosum* might employ an alternative secretory pathway, as 90.6% of the validated secretory/extracellular proteins lacked the signal peptide. A comparison of 20 oomycete genomes showed 69 *P. insidiosum*–specific secretory/extracellular proteins, and these may be responsible for the host-specific infection. The differential expression analysis revealed 14 markedly upregulated proteins (particularly cyclophilin and elicitin) at body temperature which could contribute to pathogen fitness and thermotolerance. Our search through a microbial virulence database matched 518 secretory/extracellular proteins, such as urease and chaperones (including heat shock proteins), that might play roles in *P. insidiosum* virulence. In conclusion, the identification of the secretome promoted a better understanding of *P. insidiosum* biology and pathogenesis. Cyclophilin, elicitin, chaperone, and urease are top-listed secreted/extracellular proteins with putative pathogenicity properties. Such advances could lead to developing measures for the efficient detection and treatment of pythiosis.

## 1. Introduction

*Pythium insidiosum* infects humans and animals worldwide and causes pythiosis, a devastating disease [1,2,3,4]. It is a member of the so-called oomycetes, a unique group of filamentous microorganisms that are not fungi but belong to the Phylum Oomycota of the Stramenopiles (https://www.ncbi.nlm.nih.gov/taxonomy; accessed on 14 May 2022) [1,2]. Direct exposure to a pathogen habitat (swampy areas such as paddy fields, water reservoirs, and canals) increases the risk of acquiring the infection [5,6,7,8]. Definitive diagnosis of pythiosis can be made by using various techniques (i.e., microbiological methods, serological tests, immunohistology, molecular detection, and proteomic analysis), many of which are not widely available [9,10,11,12,13,14,15,16,17,18,19,20,21,22]. Conventional antimicrobial drugs usually fail in treating pythiosis [3,4,23,24]. An affected organ (i.e., leg, arm, and eye) is typically removed to prevent disease progression and recurrence. Many patients die from aggressive and uncontrollable pythiosis [4,24].

Successful pathogens must develop a strategy to overcome host defenses to survive within their host and cause tissue damage. The secretion of virulence proteins is one of such strategies during infection. Secretory proteins have been studied to elucidate the pathogenicity of many pathogens. For example, *Blastomyces dermatitidis* secretes the virulence protein BAD1, which recombines with the fungal cell wall and serves as an adhesin [25,26]. *Cryptococcus neoformans* produces the extracellular mannoprotein Cig1 to promote virulence and iron acquisition [27,28]. In some pathogenic oomycetes (i.e., *Phytophthora ramorum* and *Phytophthora infestans*), several secreted proteins (i.e., CBEL, elicitin, protease, protease inhibitor, and Nep1-like proteins) are associated with their pathogenicity [29,30,31,32,33,34].

While most pathogenic oomycetes infect plants, *P. insidiosum* infects humans and animals [1,35,36]. As a successful pathogen, *P. insidiosum* must resist host body temperature, evade host immunity, and cause tissue destruction. Because secretory proteins are essential for microbial virulence, this type of protein might contribute to the pathogenesis of *P. insidiosum*. An investigation of this hypothesis relying solely on bioinformatic analysis of *P. insidiosum*’s genetic data [37,38,39,40,41,42,43] could result in biologically inaccurate findings. The current study employed proteogenomic analyses to identify and validate the secretory proteins (secretome) of *P. insidiosum*. We also identified species-specific, body temperature-dependent, and pathogenicity-related secretomes of this understudied pathogen. Exploring the secretory proteins of *P. insidiosum* would promote a better understanding of the pathogen’s virulence and biology and could lead to discovering a better detection and controlling method for this pathogen.

## 2. Materials and Methods

### 2.1. Microorganism and Protein Extraction

The *P. insidiosum* strain Pi-S was isolated from a Thai patient with vascular pythiosis and subjected to protein extraction for mass spectrometric analysis. The organism’s identity was confirmed by PCR and rDNA sequence analyses [14]. Extracellular proteins of *P. insidiosum* grown at two different temperatures (i.e., 25 and 37 °C) were prepared by using the established protocol [44]. In brief, *P. insidiosum* was subcultured on a Sabouraud dextrose (SD) agar (1% (*w/v*) Bacto peptone (Gibco, MI, USA), 2% (*w/v*) Glucose anhydrous (VWR Chemicals, BDH , Leuven, Belgium), and 1.5% (*w/v*) agar technical solidifying agent (Difco, MD, USA), incubated at 25 or 37 °C, and measured for colony’s radial growth (5 replicates) daily for a week. Ten pieces (5 mm in diameter) of SD agar with an actively growing *P. insidiosum* colony (7 days of age) were transferred to a 500 mL flask containing 100 mL SD broth (1% (*w/v*) Bacto peptone (Gibco, MI, USA) and 2% (*w/v*) Glucose anhydrous (VWR Chemicals, BDH, Leuven, Belgium) and incubated with shaking (150 rpm) at 25 or 37 °C for 7 days. A portion of *P. insidiosum* colony was stained with lactophenol blue solution (Merck, Darmstadt, Germany) and checked for microscopic features (i.e., hyphae) under an ECLIPSE Ci light microscope (Nikon, Tokyo, Japan; 200× magnification). The culture broth was filtered through a Durapore membrane filter (0.22 μm pore size; Millipore, Burlington, MA, USA) to remove the organism, resulting in cell-free culture filtrate antigens (CFAs). CFAs were concentrated (at least 50 folds) by centrifugation, using an Amicon Ultra-15 centrifugal filter (10,000 nominal molecular weight limit; Millipore, Burlington, MA, USA). Two biological replicates of CFAs extracted from the cultures at each temperature were prepared (4 replicates in total) and kept at −20 °C until use.

### 2.2. Protein Digestion and Liquid Chromatography–Tandem Mass Spectrometry

Protein digestion and liquid chromatography–tandem mass spectrometric analysis (LC–MS/MS) of CFA were performed, as previously described [45]. A mixture of CFA (10 µg), 2x Laemmli sample buffer (Bio-Rad, Hercules, CA, USA), and 2-mercaptoethanol was heated at 65 °C for 10 min and separated by SDS–PAGE (5% stacking and 12% resolving gels), using a Bio-Rad mini-vertical electrophoresis system. The Coomassie brilliant blue-stained SDS–PAGE gel was divided into individual lanes, each of which was cut into small blocks by using a sterile sharp blade. The gel blocks were de-stained with 50% acetonitrile in 50 mM NH_4_HCO_3_, mixed with 10 mM dithiothreitol, and incubated at 60 °C for 15 min. The sample was mixed with 55 mM iodoacetamide in 50 mM NH_4_HCO_3_ and incubated at room temperature for 30 min (protected from light). The gels were dehydrated with 100% acetonitrile (Sigma-Aldrich), digested with 0.1 mg/mL trypsin (Sigma-Aldrich) in 50 mM ammonium bicarbonate, and incubated at 37 °C overnight. After the digested proteins were solubilized by using 50% acetonitrile, the supernatant was collected and dried in a centrifugal concentrator (TOMY, Tokyo, Japan) at 45 °C.

The trypsin-treated sample (resuspended in 0.1% formic acid) was analyzed by using an Ultimate 3000 nano-LC system (Dionex, Surrey, UK) with a 300 nL/min separation flow rate and a gradient of 45 min. The reverse-phase chromatography column was the Acclaim PepMap RSLC 75 μm × 15 cm nanoviper C18, 2 μm particle size, 100 Å pore size (Thermo Scientific, Waltham, MA, USA). Mobile phase A was 2% acetonitrile and 0.1% formic acid in the water, while mobile phase B was 0.1% formic acid in acetonitrile. The eluate was analyzed by using a MicrOTOF-Q II mass spectrometer (Bruker, Karlsruhe, Germany). The Hystar software (Bruker, Dortmund, Germany) was used to acquire the data, with the survey scan mode covering the 400–2500 *m*/*z* mass range and the MS/MS spectra covering the 50–1500 *m*/*z* mass range.

### 2.3. Proteomic Data Analysis

The Compass DataAnalysis software (Bruker, Dortmund, Germany) converted the ‘.d’ files (obtained from the micrOTOF-Q II mass spectrometer; see above) to ‘.mgf’ files, which were subject to search against the genome-predicted proteome of the *P. insidiosum* strain Pi-S [39], using an in-house Mascot server version 2.3.0 (Matrix Science, London, UK). For the search setting, trypsin was set as the enzyme, one missed cleavage site was allowed, variable modifications included carbamidomethyl (C) and oxidation (M), MS peptide tolerance was 0.6 Da, MS/MS tolerance was 0.8 Da, and peptides with 95% confidence were acceptable. The exponentially modified protein abundance index (emPAI) [46,47], which is a protein quantification unit, was defined as (i) actual emPAI units (for detected and quantifiable proteins), (ii) no emPAI (for detected, but unquantifiable, proteins), or (iii) “-” (for undetected proteins).

### 2.4. Bioinformatic Analysis

A set of genome-derived *P. insidiosum* proteins with a signal peptide (*n* = 1208; predicted using the SignalP 4.0 software [48]) were obtained from the previous study [45]. To identify putative virulence proteins, LC–MS/MS-validated secretory/extracellular proteins of *P. insidiosum* were BLAST-searched against the MvirDB database [49]. Functional annotation was performed by BLAST searches of the secretory/extracellular proteins against the Clusters of Orthologous Groups of Proteins (COGs) database [50,51]. Proteins were grouped and compared by using Microsoft Excel for Mac (version 16.29) and InteractiVenn [52] programs. Selected proteins were subject to (i) BLASTP search against the NBCI non-redundant protein database (https://blast.ncbi.nlm.nih.gov/Blast.cgi, accessed on 15 March 2022); and (ii) conserve domain search [53,54], using the Web CD-search tool with the default setting (https://www.ncbi.nlm.nih.gov/Structure/cdd/wrpsb.cgi, accessed on 15 March 2022).

### 2.5. Data Availability

The draft genome sequence is available in the DDBJ/NCBI databases through the accession numbers BBXB01000001–BBXB01001192. The LC–MS/MS-derived peptide data were deposited into the ProteomeXchange Consortium via the PRIDE [55] partner repository with the dataset identifier PXD031111.

## 3. Results

### 3.1. Identification and Validation of P. insidiosum Secretome

The draft genome sequence of the *P. insidiosum* strain Pi-S was obtained from the NCBI/DDBJ database [39]. In this genome, 14,962 open reading frames were identified and deduced to protein sequences, 8.1% (*n* = 1208) of which contained a signal peptide predicted by the SignalP software [39,45,48]. These predicted proteins were in silico cleaved by trypsin into multiple peptides and served as an in-house Mascot database. Four biological replicates of *P. insidiosum*’s CFA (representing the crude secretory/extracellular proteins) were prepared from hyphae-removed liquid cultures incubated at 25 °C (two replicates: CFA25-1 and CFA25-2) and 37 °C (two replicates: CFA37-1 and CFA37-2) for shotgun LC–MS/MS analysis. The identification and validation of the *P. insidiosum* secretory/extracellular proteins adopted the criteria of the previously reported protocol using the LC–MS/MS-derived data [45]. A peptide sequence detected by the LC–MS/MS analysis in two or more CFA replicates was recruited for validating all genome-derived 14,962 proteins of *P. insidiosum*. As a result, 2980 predicted proteins (19.9%) can be mapped with at least two recruited CFA-derived LC–MS/MS-generated peptides and assigned as validated secretory/extracellular proteins. 

**Figure 1:**
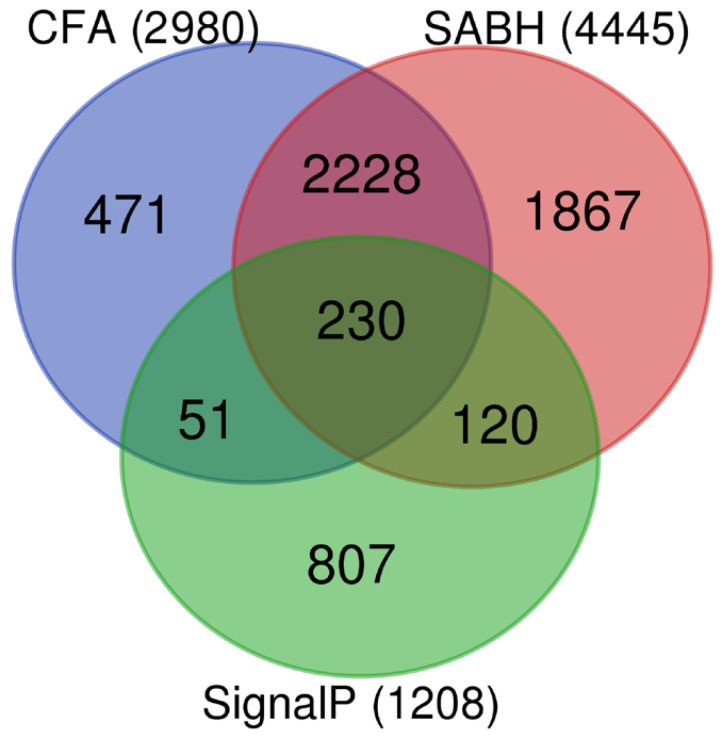
Venn diagram of the validated secretory/extracellular (*n* = 2980), cytosolic/intracellular (*n* = 4445), and SignalP-positive (*n* = 1208) proteins of *P. insidiosum*. CFA (culture filtrate antigens) and SABH (soluble antigens from broken hyphae) sets represent secretory/extracellular and cytosolic/intracellular proteins in origins, respectively. The SignalP set includes proteins that contain a signal peptide predicted by the SignalP software.

Of 14,962 proteins predicted in the *P. insidiosum* genome, 4445 (29.7%) were previously validated as cytosolic/intracellular proteins by LC–MS/MS analysis of soluble antigens from broken hyphae (SABH; representing cytosolic/intracellular proteins) [45]. Proteins from the secretory/extracellular set (*n* = 2980), cytosolic/intracellular set (*n* = 4445), and the SignalP-positive set (*n* = 1208) were compared for similarities and differences by using a Venn diagram (Figure 1). In total, 4967 (33.2%) of 14,962 predicted proteins were confirmed their expressions in *P. insidiosum* by the CFA- and SABH-derived LC–MS/MS data. Of these 4967 validated proteins, 522 (10.5%), 1987 (40.0%), and 2458 (49.5%) were present in only extracellular space, only intracellular space, and both spaces, respectively. From 1208 proteins with a predicted signal peptide (SignalP-positive proteins), only 281 (23.3%) can be validated as secretory/extracellular proteins. Moreover, most validated secretory/extracellular proteins (*n* = 2699; 90.6%) lacked a predicted signal peptide (SignalP-negative proteins).

The Oomycete Gene Table (available online at https://sbi.pdti.kmutt.ac.th/cgi-bin/gt/viewer?organism=oomycetes&build=150418, accessed on 15 March 2022) was used to compare the genome contents of 20 different oomycetes (including *P. insidiosum*, six non-*insidiosum Pythium* species, six *Phytophthora* species, two *Albugo* species, two *Aphanomyces* species, two *Saprolegnia* species, and *Hyaloperonospora arabidopsis*) and two diatoms (including *Phaeodactylum tricornutum* and *Thalassiosira pseudonana*) [45,56]. A set of 1194 *P. insidiosum*–specific proteins were identified, all of which had no defined function [45]. The Venn diagram analysis showed that 69 (5.8%) of the *P. insidiosum*–specific proteins were validated secretory/extracellular proteins.

### 3.2. Functional Annotation of P. insidiosum’s Secretome

**Figure 2:**
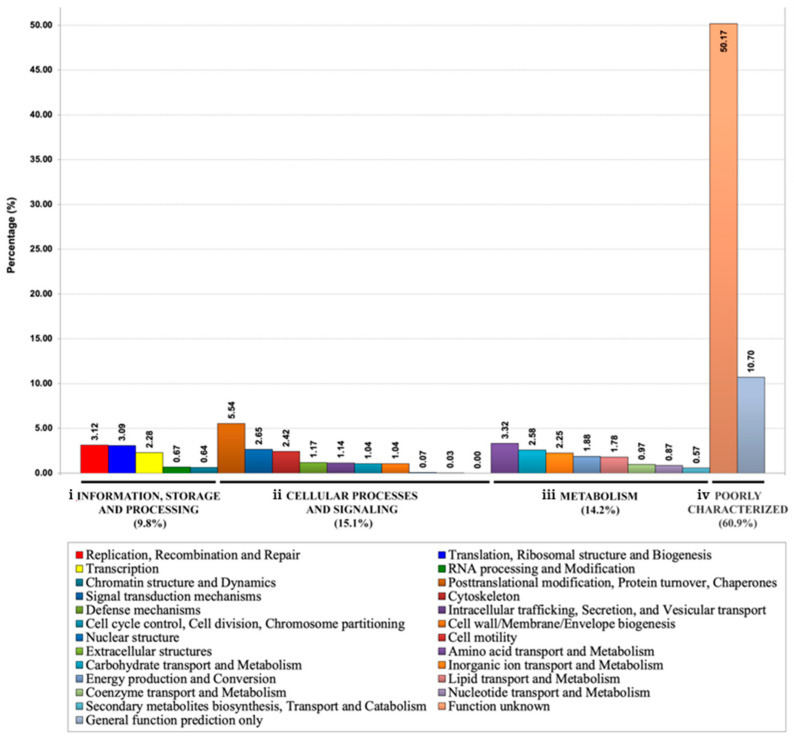
Classification of the *P. insidiosum* secretory/extracellular proteins based on the Clusters of Orthologous Groups of Proteins (COG) database. All 2980 validated secretory/extracellular proteins of *P. insidiosum* are allocated into 4 primary COG groups: (**i**) information storage and processing (*n* = 292; 9.8%; consisting of 5 subgroups); (**ii**) cellular processes and signaling (*n* = 450; 15.1%; 10 subgroups); (**iii**) metabolism (*n* = 424; 14.2%; 8 subgroups); and (**iv**) poorly characterized function (*n* = 1814; 60.9%; 2 subgroups). The box shows all 25 COG-defined functional subgroups.

Based on the BLAST search against the COG database [50,51], 2980 validated secretory/extracellular proteins of *P. insidiosum* were assigned to four primary COG categories: (i) information storage and processing (*n* = 292; 9.8%), (ii) cellular processes and signaling (*n* = 450; 15.1%), (iii) metabolism (*n* = 424; 14.2%), and (iv) poorly characterized function (*n* = 1814; 60.9%) (Figure 2). When subcategorizing these secretory/extracellular proteins into 25 COG-defined functional groups; 50.2% (*n* = 1495) fell into unknown function, 10.7% (*n* = 319) showed a non-specific function prediction; and 39.1% (*n* = 1166) resided in 22 out of 23 well-characterized functional groups, such as posttranslational modification, protein turnover, chaperones (*n* = 165; 5.5%); amino acid transport and metabolism (*n* = 99; 3.3%); replication, recombination, and repair (*n* = 93; 3.1%); translation, ribosomal structure, and biogenesis (*n* = 92; 3.1%); signal transduction mechanisms (*n* = 79; 2.7%); and carbohydrate transport and metabolism (*n* = 77; 2.6%) (Figure 2).

### 3.3. Effect of the Temperature on the Growth of the Organism and Expression of the Secretory/Extracellular Proteins

**Figure 3:**
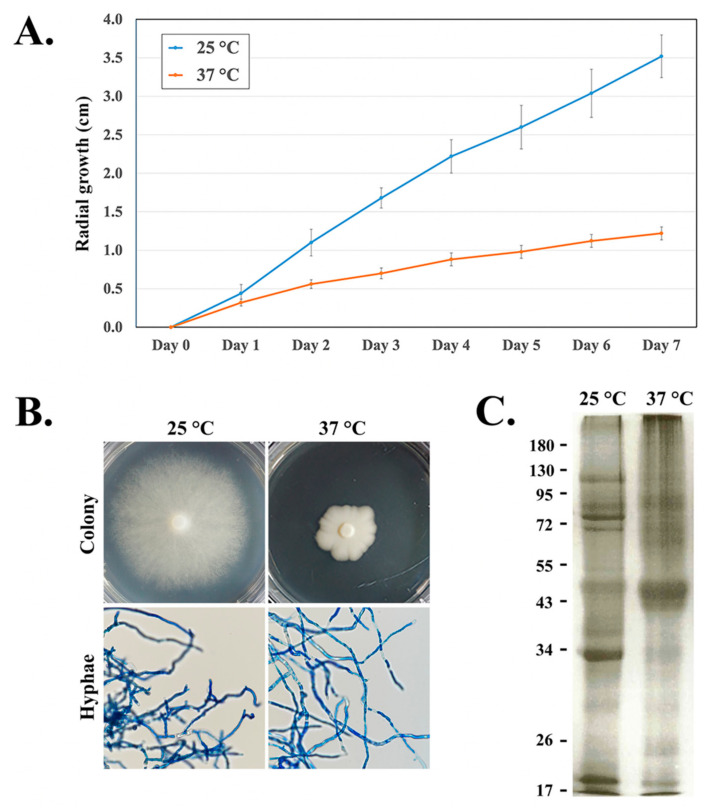
Effect of the temperatures (25 vs. 37 °C) on the growth and the secretory/extracellular protein expression of *P. insidiosum*. The organism incubated at a different temperature shows (**A**) radial growth curve, (**B**) colony density, and (**C**) secretory/extracellular protein profile (demonstrated by SDS-PAGE analysis; molecular weight marker range, 17–180 kilodaltons) during the 7-day course. The microscopic features (branching hyphae) of the pathogen grown at either temperature condition were recorded by using an ECLIPSE Ci light microscope (Nikon, Tokyo, Japan; 200× magnification; B).

During the 7-day incubation, *P. insidiosum* exposed to 37 °C (body temperature) showed a dense colony and relatively reduced radial growth compared with that of the organism exposed to 25 °C (ambiance temperature) (Figure 3A,B). However, the organism’s microscopic features (branching hyphae) at both temperatures were similar (Figure 3B). After the extracted secretory/extracellular proteins from *P. insidiosum* that were incubated at a different temperature were separated by SDS–PAGE, different protein profiles were observed (Figure 3C): some protein sizes (such as 43–50, 60–72, and 90–95 kDa) were abundant at 37 °C, while several proteins (such as 34, 74, 80, and 120 kDa) were prominent at 25 °C.

The effect of the different temperatures (ambiance vs. body temperature) on protein expressions in *P. insidiosum* was determined. Abundances of secretory/extracellular proteins when the pathogen in vitro exposed to 25 and 37 °C (analyzed in duplicate for each temperature; sample IDs, CFA25-1, CFA25-2, CFA37-1, and CFA37-2) were compared based on the LC–MS/MS data generated from the hyphae-removed liquid cultures. An averaged protein abundance was reported by using the emPAI unit (see the Methods section). If a protein was assigned as “no emPAI” (representing a detected non-quantifiable protein) in at least one biological replicate, it was excluded from differential protein expression analysis. In total, 192 quantifiable proteins passed the following criteria: 26.0% (*n* = 50 proteins) at body temperature (37 °C), 16.7% (*n* = 32) at ambient temperature (25 °C), and 57.3% (*n* = 110) at both conditions.

**Figure 4:**
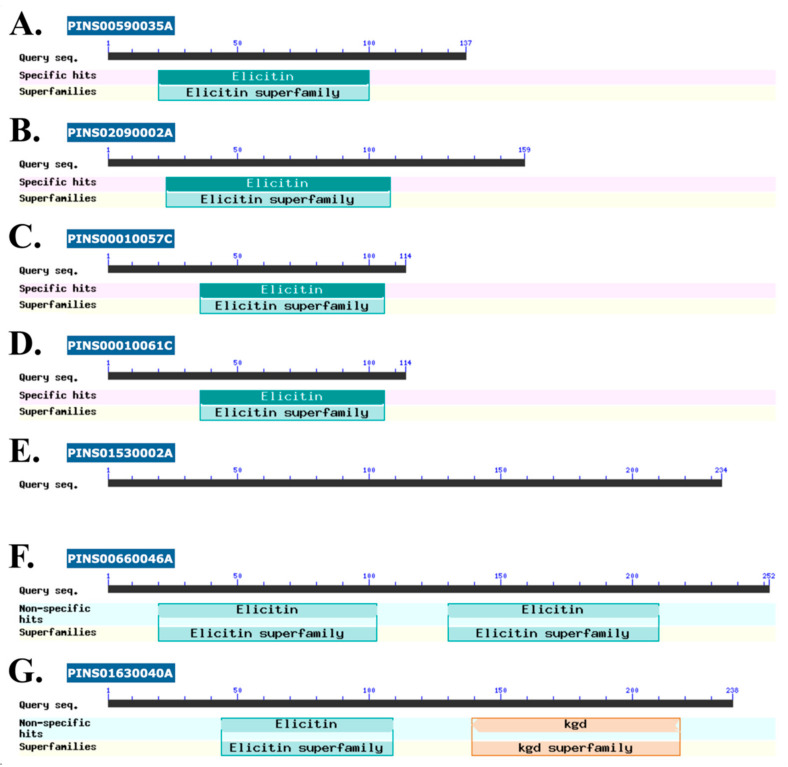
Functional domains present in the body temperature-upregulated proteins of *P. insidiosum*. The Web CD-search tool (https://www.ncbi.nlm.nih.gov/Structure/cdd/wrpsb.cgi; accessed on 1 March 2022) is used to detect the protein domain. Elicitin-like (**A**,**B**) and hypothetical (**C**,**D**,**F**,**G**) proteins contain 1 or 2 elicitin domains (green boxes). The hypothetical protein PINS01530002A lacks a defined functional domain (**E**). One hypothetical protein (PINS01630040A) harbors elicitin and kgd (orange box) domains (**G**). The blue boxes show protein IDs (as detailed in Table 1). The black straight line demonstrates the relative protein length with numbers indicating the amino acid positions in each protein.

The criteria for selecting markedly upregulated extracellular proteins at the body temperature were as follows: (i) protein was only detected at 37 °C with an average protein abundance greater than 1 emPAI; or (ii) 37 to 25 °C relative protein abundance was greater than five times. The top 14 upregulated proteins at the body temperature included five hypothetical (uncharacterized) proteins, two elicitin-like proteins, two cofilin/tropomyosin-type actin-binding proteins, cyclophilin A, NAD-dependent malate dehydrogenase, mitochondrial ATP synthase subunit beta, glycerophosphoryl diester phosphodiesterase, and glycoside hydrolase (Table 1). Among these proteins, three proteins exhibited extremely high protein abundance at 37 °C: cyclophilin A (length, 171 amino acids; abundance, 50.2 emPAI; accession, GAY01457.1), elicitin-like protein PINS02090002A (159 amino acids; 72.0 emPAI; GAY02672.1), and elicitin-like protein PINS00590035A (137 amino acids; 9.9 emPAI; GAX97068.1). A conserved domain search [53,54] detected one “elicitin” domain in two elicitin-like and all hypothetical proteins (Figure 4), except the protein PINS00660046A, which had two such domains (Figure 4F), and PINS01530002A, which had no defined domain (Figure 4E). Two elicitin domain-containing hypothetical proteins (PINS00010057C and PINS00010061C) shared the same length (114 amino acids long) with high (96%) sequence identity (Figure 4C,D). One hypothetical protein (PINS01630040A) harbored two different domains: elicitin and kgd (Figure 4G). Of these 14 markedly upregulated proteins, eight (five hypothetical proteins, two elicitin-like proteins, and a glycoside hydrolase) had no match with any sequences in the NCBI non-redundant human protein database (Table 1).

**Table 1:** Top upregulated secretory/extracellular proteins (*n* = 14) of *P. insidiosum* upon exposure to the body temperatures (37 °C).

*P*. *insidiosum* Protein ID	Functional Annotation ^a^	Average Protein Abundance at 37 °C (emPAI ^b^)	Average Protein Abundance at 25 °C (emPAI)	Relative Protein Abundance (37:25 °C)	Accession Number
PINS01640001A	Cyclophilin A	50.18	-	-	GAY01457.1
PINS00590035A	Elicitin-like protein ^c,d^	9.94	-	-	GAX97068.1
PINS00010057C	Hypothetical protein ^c,d^	6.69	-	-	GAX92118.1
PINS00010061C	Hypothetical protein ^c,d^	3.50	-	-	GAX92125.1
PINS01530002A	Hypothetical protein ^d^	1.79	-	-	GAY01151.1
PINS00660046A	Hypothetical protein ^c,d^	1.69	-	-	GAX97463.1
PINS00380039A	ATP synthase subunit beta, mitochondrial	1.44	-	-	GAX95749.1
PINS00660004A	Glycerophosphoryl diester phosphodiesterase	2.34	0.22	10.61	GAX97472.1
PINS02090002A	Elicitin-like protein ^c,d^	71.97	7.82	9.21	GAY02672.1
PINS01630040A	Hypothetical protein ^c,d^	1.27	0.15	8.43	GAY01431.1
PINS02430017C	Cofilin/tropomyosin-type actin-binding protein	0.11	0.02	7.33	GAY03393.1
PINS00560010C	Cofilin/tropomyosin-type actin-binding protein	0.14	0.02	7.00	GAX96878.1
PINS00420001A	Malate dehydrogenase, NAD-dependent	2.00	0.30	6.76	GAX96042.1
PINS06030002B	Glycoside hydrolase ^d^	0.11	0.02	5.25	GAY06261.1

^a^ BLASTP search against the NCBI non-redundant protein database. ^b^ The protein quantification unit is defined as the exponentially modified protein abundance index, or emPAI. ^c^ Conserve domain search using the Web CD-search tool (https://www.ncbi.nlm.nih.gov/Structure/cdd/wrpsb.cgi; accessed on 1 March 2022) [53,54] identifies 1 or 2 “elicitin” domains. ^d^ No homologous sequence was found in the NCBI non-redundant human protein database.

### 3.4. Putative Pathogenicity Proteins in P. insidiosum’s Secretome

Based on BLAST search against the MvirDB database (a microbial virulence factor collection) [49], 518 (17.4%) of all 2980 LC–MS/MS-validated secretory/extracellular proteins of *P. insidiosum* hit 221 unique virulence factor identification numbers (VFID) assigned to pathogenicity-related proteins of other pathogens. Based on *E*-values, the top 20 VFIDs are listed in Table 2, and they include heat shock protein (HSP) 70, HSP90, urease, chaperone protein ClpB, carbamoyl phosphate synthetase II, tripeptidyl-peptidase, eukaryotic translation initiation factor, chaperonin GroEL, phosphoglucomutase, and transcription factor site-1 protease. Of these MvirDB-matched proteins, only urease (PINS00920015C) showed no homologous sequence in the NCBI human protein database.

## 4. Discussion

In this study, we sought secretory/extracellular proteins of *P. insidiosum* as a part of the attempts to elucidate the biology and pathogenesis of this understudied but highly virulent microorganism. The shotgun LC–MS/MS analysis of CFA (representing secretory/extracellular proteins) and SABH (representing cytosolic/intracellular proteins) confirmed the expressions of only one-third (33.2%; *n* = 4967) of the 14,962 proteins predicted in the *P. insidiosum* genome. The proteins presented only in SABH (*n* = 1987; 40.0% of 4967 LC–MS/MS-validated proteins) should function inside the cell, whereas a smaller part presented only in CFA (*n* = 522; 10.5%) is likely to be secreted, accumulated, and functioning outside (Figure 1). The proteins identified in both CFA and SABH (*n* = 2458; 49.5%) could function intracellularly and extracellularly or result from cell lysis (Figure 1). However, the hyphal culture used to prepare CFA was young (~7 days old), suggesting that cell autolysis was unlikely. In total, 2980 proteins were successfully validated to be secreted or translocated to the extracellular space. While the majority (60.9%) of these validated secretory/extracellular proteins had poorly characterized or unknown function, the rest were bioinformatically predicted to involve in various cellular processes, including information storage and processing (9.8%), cellular processes and signaling (15.1%), and metabolism (14.2%) (Figure 2).

SignalP, a well-known and popular bioinformatics software [48], predicted a total of 1208 secretory proteins in *P. insidiosum* [39,45]. The software relies on in silico analysis of protein sequences to define a signal peptide (at the N-terminus) that guides the protein through the classical secretory pipeline (involving the endoplasmic reticulum and the Golgi apparatus) in eukaryotes [57]. The prediction accuracy of SignalP in oomycetes, particularly *P. insidiosum*, is unknown. As shown here, SignalP detected a signal peptide only in 281 (9.4%) out of 2980 LC–MS/MS-verified secretory/extracellular proteins of *P. insidiosum* (Figure 1), indicating that most validated proteins (90.6%; *n* = 2699) lacked a predicted signal peptide. Moreover, SignalP also assigned a signal peptide to 120 (6.4%) out of 1867 LC–MS/MS-verified proteins presented only inside the cells (Figure 1), suggesting a limited detection specificity of the software. Thus, using and interpreting a SignalP-predicted protein should be cautious for a possible inaccurate result. Secretion of those SignalP-negative proteins may depend on an alternative secretory pathway, such as direct translocation, lysosomal secretion, exosome-dependent secretion, and plasma-membrane blebbing and shading [57].

In contrast to most other pathogenic oomycetes, which cause disease in plants, *P. insidiosum* causes a high mortality and morbidity infection in both humans and animals [1,36]. During its evolution, *P. insidiosum* might have established a set of unique proteins for a host-specific infection. As secretory proteins contribute to the pathogenicity of many pathogens [25,26,27,28,29,30,34], we focused on searching *P. insidiosum*–specific secretory/extracellular proteins by using the Oomycete Gene Table for comparative analysis of 20 oomycete genomes [45,56], and, as a result, we found 69 such proteins. Although their functions are unknown, these unique proteins may be involved in pythiosis pathogenesis in humans and animals. The *P. insidiosum*–specific secretory/extracellular proteins could be clinically applied as a circulating marker for definitive diagnosis and treatment monitoring when detecting the anti-pathogen antibodies is not helpful, especially in a patient with recurrent infection or immunotherapy. Genome data availability and cell-free protein synthesis technology make it possible to identify the coding sequences and facilitate the rapid protein production, screening, and characterization for such biological markers [39,40,41,42,58].

**Figure 5:**
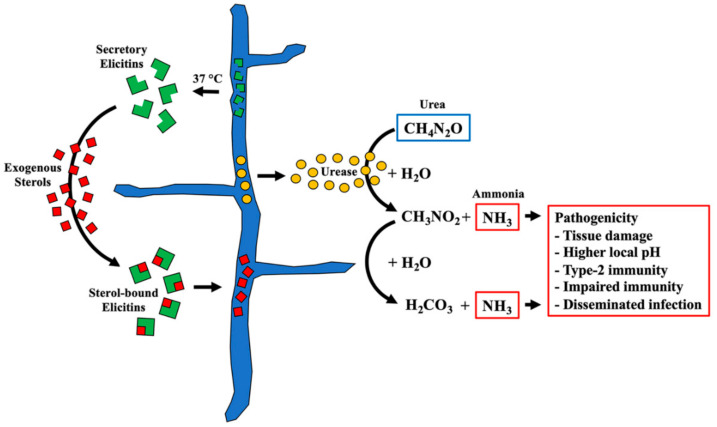
Proposed pathogenicity model of *P. insidiosum*. The pathogen secretes an array of virulence proteins to survive stress conditions and establish an infection inside the host. For example, on the left-hand side, a set of diverse elicitins released from *P. insidiosum* hyphae upon exposure to body temperature (37 °C) compete with the host in acquiring exogenous sterols (a major cell membrane component unable to synthesize by the pathogen) for microbial growth and fitness. On the right-hand side, the enzyme urease presented inside and outside the pathogen hydrolyzes urea (an amino acid breakdown product generated throughout the host body) to obtain carbamic acid (CH_3_NO_2_), carbonic acid (H_2_CO_3_), and ammonia (NH_3_). Ammonia could mediate several pathogenic features in the host, such as cell/tissue damage, increased local pH, Type-2 immunity polarization, impaired immune function (i.e., phagocytic activity), and disseminated infection.

One of the microbial abilities to cause infection in humans and animals is resistance to increased temperatures inside hosts. While most *Pythium* species have optimal growth at 25–30 °C, only a few species, notably *P. insidiosum* and *Pythium aphanidermatum* (known human/animal pathogenic species), grow well at 35–40 °C or even higher [35,59,60,61,62]. The ambient temperature at which *P. insidiosum* grows in the environment is around 25 °C. The changed growth, colony density, and protein profiles suggested that *P. insidiosum* adapted to an increased temperature (Figure 3). Exploring the proteins upregulated in response to the transition from ambient (25 °C) to body (37 °C) temperature might give a clue into the thermotolerant mechanism and virulence of *P. insidiosum*. Thus, we compared 2 sets of LC–MS/MS-validated secretory/extracellular proteins from *P. insidiosum* exposed to 25 °C and 37 °C. Based on stringent criteria, 14 (7.3%) out of 192 quantifiable proteins (especially cyclophilin A and elicitin domain-containing proteins) markedly increased their expressions (Table 1 and Figure 4), implying that these upregulated proteins might be linked to the high-temperature adaptation and pathogenesis of the pathogen. Cyclophilins are conserved among eukaryotes and prokaryotes and are involved in various biological and pathogenicity-related processes, such as protein folding, signaling, transcription/translation regulation, cell cycle and replication, stress response, and microbial virulence [63]. The deletion of cyclophilin in phytopathogens and *Burkholderia pseudomallei* reduces pathogenicity [64]. In oomycetes (i.e., *Phytophthora* species), cyclophilins are markedly expressed during infection [65]. Regarding elicitins, they form a protein family only presented in oomycetes and might play a role in acquiring exogenous sterols (crucial cell membrane components), promoting infection, and inducing host plant immunity [66,67,68,69,70,71,72]. Because *P. insidiosum* lacks a complete sterol biosynthesis pathway, the organism expresses a set of diverse elicitins, and homology modeling suggests the elicitins can encapsulate a sterol (i.e., cholesterol or ergosterol) [37,38,45,71], it has been proposed that the pathogen secretes an array of elicitins to function as sterol-carrier proteins in competing with the host to obtain exogenous sterols (Figure 5). Elicitins could evade host antibody recognition [72], allowing them to function in the host uninterruptedly. Moreover, multiple elicitins were simultaneously upregulated at body temperature (Table 1), emphasizing their potential contribution to biological fitness and pathogenesis, as well as possible clinical applications, such as serving as a diagnostic marker.

The validated secretory/extracellular proteins of *P. insidiosum* matched various microbial virulence-related proteins (assigned as VFIDs) in the MvirDB database [49] (Table 2). Among the top MvirDB-matched proteins, chaperones (including heat shock proteins) and urease identified in *P. insidiosum* became our focus because the homologous proteins described in other pathogens exhibit profound virulence characteristics. The chaperones are quite conserved among living cells and are essentially involved in protein folding/maturation, as well as the invasion, infection, and stress responses of many pathogenic viruses, bacteria, parasites, and fungi [73,74,75,76,77,78]. In *Cryptococcus neoformans*, HSP90 is secreted to localize on the cell surface, and it mediates thermotolerance, capsule formation, and antifungal drug resistance [74]. Some parasites employ a heat shock protein for stage conversion of, for example, the insect form to mammalian form in *Leishmania donovani*, the blood form trypomastigotes to epimastigotes in *Trypanosoma cruzi*, and the bradyzoite to tachyzoite stage in *Toxoplasma gondii* [75]. *P. insidiosum* also undergoes stage conversion from the motile biflagellate zoospore to invasive hyphae in the host. The pathogen might adopt some of these pathogenicity mechanisms to the identified chaperones (i.e., HSP70, HSP90, and chaperones ClpB and GroEL; see Table 2) during the pathogenesis of pythiosis.

Regarding urease, it is a well-recognized virulence determinant of some pathogens, such as *Helicobacter pylori* and *Cryptococcus neoformans* [79,80,81]. *Helicobacter pylori* has adapted itself to grow in the low-pH environment of the gastrointestinal tract by expressing a high level of urease for hydrolyzing host urea (a breakdown product of amino acids) to form toxic ammonia, resulting in increased local pH and cell destruction (i.e., epithelial cell) at the infection site [80,82,83]. *Cryptococcus neoformans* also expresses urease to promote host immune evasion, cell damage, and disseminated infection [80]. Urease activity is associated with the unprotective Type-2 immunity against *Cryptococcus neoformans* infection, rather than the Type-1 immunity, which is considered protective against this pathogenic fungus [80]. A urease-deficient strain of *Cryptococcus neoformans* shows markedly attenuated virulence in the mouse model [79]. Ureases of *P. insidiosum* and *Cryptococcus neoformans* share a high degree of sequence similarity (71%) and identity (57%) [45]. *P. insidiosum* might adopt the pathogenic properties of urease from those pathogens as a part of its ability to infect the gastrointestinal tract, arteries, skin, eye, and multiple organs (in disseminated infection) [4] (Figure 5). The urease (Ure1) of *P. insidiosum* has been detected inside (previous report [45]) and outside (this study) the cell, implying that the enzyme could function both intracellularly and extracellularly. Besides its profound virulence effects, a urease-coding homologous sequence is not present in humans, making this enzyme an appealing diagnostic marker and therapeutic target.

In conclusion, the secretion of an array of virulence proteins during infection is one of the pathogen strategies to survive, combat, and cause tissue destruction within hosts. Knowing a set of secreted/extracellular proteins, so-called secretome, could promote a better understanding of microbial biology and pathogenesis. Here, we employed the proteogenomic analysis of proteomic and genomic data to comprehensively profile a secretome, consisting of the species-specific, temperature-dependent, and putative virulence proteins of *P. insidiosum*. Cyclophilin, elicitin, chaperone, and urease were at the top of the list of those validated as secreted/extracellular proteins that have potential pathogenicity-associated properties. Not only do these findings help us better recognize biological and pathological processes, but such advances could also lead to developing measures for the efficient detection and treatment of the infection caused by *P. insidiosum*.

## Figures and Tables

**Table 2:** The top 20 virulence factor identification numbers (VFIDs) of the MvirDB database that best match LC–MS/MS-validated secretory/extracellular proteins of *P. insidiosum*.

VFID	MvirDB-Defined Virulence Factors	Organisms	Virulence Types	*P*. *insidiosum* Protein ID	Accession Number	Identity (%)	E-Value
26381	Heat shock protein 70	*Cryptosporidium parvum*	Virulence protein	PINS00530003B	GAX96752.1	71.24	0
26468	Organellar heat shock protein	*Eimeria tenella*	Virulence protein	PINS01150027A	GAX99732.1	65.0	0
12025	Urease	*Oryza sativa*	Virulence protein	PINS00920015C ^a^	GAX98700.1	64.5	0
15128	Chaperone protein ClpB	*Francisella tularensis*	Virulence protein	PINS01620006A	GAY01393.1	55.1	0
26434	Carbamoyl phosphate synthetase II	*Toxoplasma gondii*	Virulence protein	PINS04750002A	GAY05754.1	53.3	0
11169	Tripeptidyl-peptidase 2	*Mus musculus*	Protein toxin	PINS01420022C	GAY00792.1	34.8	0
26447	Heat shock protein 70	*Toxoplasma gondii*	Virulence protein	PINS00020096A	GAX92393.1	57.6	0
26455	Eukaryotic translation initiation factor 4A	*Toxoplasma gondii*	Virulence protein	PINS00130063A	GAX93656.1	72.9	1 × 10^−173^
8350	Chaperonin GroEL	*Legionella pneumophila*	Virulence protein	PINS00020065A	GAX92364.1	57.0	3 × 10^−159^
8728	Phosphoglucomutase	*Brucella melitensis*	Virulence protein	PINS00550030A	GAX96838.1	50.4	7 × 10^−158^
12413	Transcription factor site-1 protease	*Homo sapiens*	Protein toxin	PINS02120014C	GAY02722.1	40.2	5 × 10^−142^
7573	Nonribosomal peptide synthetase Dhbf	*Bacillus anthracis*	Virulence protein	PINS01020025B	GAX99146.1	29.0	1 × 10^−113^
20164	Asparaginyl-tRNA synthetase	*Salmonella enterica*	Pathogenicity island	PINS00170005A	GAX93976.1	46.7	6 × 10^−113^
13372	Pyruvate kinase	*Salmonella typhimurium*	Virulence protein	PINS00750004A	GAX97930.1	45.9	3 × 10^−107^
26400	cGMP dependent protein kinase	*Toxoplasma gondii*	Virulence protein	PINS00410016C	GAX95955.1	33.0	6 × 10^−105^
26980	AP65-1 adhesin	*Trichomonas vaginalis*	Virulence protein	PINS01980002A	GAY02392.1	37.9	5 × 10^−101^
26382	Heat shock protein 90	*Cryptosporidium parvum*	Virulence protein	PINS00460042A	GAX96321.1	42.1	3 × 10^−100^
26459	Peroxisomal catalase	*Toxoplasma gondii*	Virulence protein	PINS00770005C	GAX98049.1	40.6	2 × 10^−92^
20579	Recombination factor protein RarA	*Salmonella enterica*	Pathogenicity island	PINS00070081C	GAX93094.1	44.8	5 × 10^−91^
14026	Lysil-tRNA synthetase LysU	*Escherichia coli*	Virulence protein	PINS00380044C	GAX95759.1	38.5	5 × 10^−91^

^a^ No homologous sequence was found in the NCBI non-redundant human protein database.

## Data Availability

The draft genome sequence is available in the DDBJ/NCBI databases through the accession numbers BBXB01000001–BBXB01001192. The LC–MS/MS-derived peptide data were deposited into the ProteomeXchange Consortium via the PRIDE [55] partner repository with the dataset identifier PXD031111.
